# Hydrogen adsorption on fcc metal surfaces towards the rational design of electrode materials

**DOI:** 10.1038/s41598-024-71703-w

**Published:** 2024-09-09

**Authors:** Cláudio M. Lousada, Atharva M. Kotasthane

**Affiliations:** Department of Materials Science and Engineering, KTH Royal Institute of Technology, SE-100 44 Stockholm, Sweden

**Keywords:** Electrocatalysis, Electronic structure, Heterogeneous catalysis

## Abstract

The successful large-scale implementation of hydrogen as an energy vector requires high performance electrodes and catalysts made of abundant materials. Rational materials design strategies are the most efficient means of reaching this goal. Here we present a study on the adsorption of H-atoms onto fcc transition metal surfaces and propose descriptors for the rational design of electrodes and catalysts by means of correlations between fundamental properties of the materials and among other properties, their experimentally measured performance as hydrogen evolution electrodes (HEE). A large set of quantum mechanical modelling data at the DFT level was produced, covering the adsorption of H-atoms onto the most stable surfaces (100), (110) and (111) of: Ag, Au, Co, Cu, Ir, Ni, Pd, Pt and Rh. For each material and surface, a coverage dependent set of minimum energy structures was produced and chemical potentials for adsorption of H-atoms were obtained. Averaging procedures are here proposed to approach modelling to the experiments. Several correlations between the computed data and experimentally measured quantities are done to validate our methodology: surface plane dependent adsorption energies, chemical potentials and experimentally determined surface energies and work functions. We search for descriptors of catalytic activity by testing correlations between the DFT data obtained from our averaging procedures and experimental data on HEE performance. Our methodology allows us to obtain linear correlations between the adsorption energy of H-atoms and the exchange current density (*i*_*0*_) in a HEE, avoiding the volcano-like plots. We show that the chemical potential has limitations as a descriptor of *i*_*0*_ because it reaches an early plateau in terms of *i*_*0*_. Simple quantities obtained from database data such as the first stage electronegativity (χ) as devised by Mulliken has a strong linear correlation *i*_*0*_. With a quantity we denominate modified second-stage electronegativity (χ_2m_) we can reproduce the typical volcano plot in a correlation with *i*_*0*_. A theoretical and conceptual framework is presented. It shows that both χ and χ_2m_, that depend on the first ionization potential, second ionization potential and electron affinity of the elements can be used as descriptors in rational design of electrodes or of catalysts for hydrogen systems.

## Introduction

Dissociative hydrogen adsorption or H-atom adsorption at metal surfaces is a phenomenon that determines the performance of innumerous systems of high technological and scientific importance. The most obvious connection between this atomic scale process with practical applications is within hydrogen production and usage. This encompasses an array of electrocatalytic systems such as electrodes in diverse electrolysis reactors^1^ or in fuel cells^2^, but also photocatalytic systems where there has been a considerable effort to develop H_2_(g) production from the sunlight assisted dissociation of H_2_O at the surface of semiconductors doped with metallic inclusions that function as co-catalysts^3,4^. H-atom adsorption is also a key parameter in corrosion and hydrogen absorption and embrittlement by metals^5,6^. Additionally, diverse important catalytic processes involve H-atom transfer reactions between molecular species at the surface of high-performance catalysts^7^.

Rational materials design is a strategy for the development of new functional, high-performance materials that is gaining momentum because of the link it can provide between properties and function^8^. This topic has attracted considerable attention because of the developments in modern experimental and computational techniques that allow more detailed studies of materials at the atomic scale^9^. To correlate atomic scale properties with processes to generate descriptors of performance for usage in rational design is of utmost importance for the successful development of better materials^10^. Improving the materials used in high performance applications is an important task that requires access to high-quality data that can be rationalized and correlated to produce descriptors for the rational design of those materials^11^. Especially relevant to this task, is to first develop correlations between properties and function for simpler materials—single component with simpler geometries—before embarking on the study of complex multicomponent-multi-structure systems^12,13^.

Despite the relevance of H-atom adsorption at metal surfaces, there is no account that systematically investigates the effects of the substrate in terms of both surface planes and increasing coverage of H-atoms on the adsorption energies (Δ*E*_ads_) and on the driving force for adsorption. Understanding these effects and the origin for the driving force for adsorption and for the formation of chemical bonds is essential to understand and predict performance of materials in real systems. The success of the *d* band model for this task is unquestionable^14,15^. However, this model requires a rather complex analysis to be put in practice^16^. Because of its inherent complexity, its physical significance may not be directly understood by researchers more oriented towards engineering that focus on system design and upscaling. For these tasks, the existence of simpler descriptors of performance that can be used in rational materials design and in the first stages of materials selection, that can link simple properties with performance, and that can be understood in terms of simple quantities that are intrinsic to the materials is important^12,17,18^.

The available bulk literature data on H-atom adsorption onto different surfaces of common transition metals has been obtained with different methods and by different authors often under different conditions as detailed in the Supplementary Information section to this paper. This has an impact especially in the computational data whose quality relies on the cancelation of errors of differential quantities such as Δ*E*_ads_. Computational data obtained with different computational methodologies has different error magnitudes and deviates from the real values in different directions—some methods systematically underestimate while others overestimate—which results in large data dispersions that do not allow accurate analysis, rationalization, and derivation of performance descriptors. Supplementary Information Figure S1 shows how Δ*E*_ads_ data for adsorption of H-atoms on Cu as a function of surface coverage defined as fraction of monolayer (ML), varies depending on the data source.

In general, for transition metals with fcc structure, the low Miller index surfaces (100), (110), and (111) are the most stable and most frequently occurring in polycrystals^19^. For practical applications these are thus the most relevant surfaces in terms of the performance of the systems where these materials are used for their surface properties. For hydrogen systems that depend on surface reactions, the adsorption energy of the hydrogen atom (Δ*E*_ads_H) is among the most significant parameters to consider. This quantity depends on both the geometrical and electronic properties of the adsorbent surfaces. In general, the traditional decoupling of geometric and electronic effects in adsorption can be seen as a necessary oversimplification, because the electronic structure of the surfaces depends on their geometries and vice-versa. Properties that depend on the surface plane such as work function (*W*) among others, are both determined by the surface structure and by bulk properties such as the ionization potential (*IP*), electron affinity (*EA*) and consequently by the electronegativity (*χ*). We have previously shown that for transition metal and lanthanide oxides in solution, very good correlations between experimentally measured catalytic activity and computational data could be obtained against the *IP*, *EA* and *χ* of the cations. These correlations could predict catalytic properties such as energy barriers and strength of adsorption of products and intermediates in processes where free radicals were generated at the surfaces^12^. Based on this principle, we have further derived a descriptor of O-atom adsorption onto clean and doped Al surfaces with different dopants. The descriptor is a modified Mulliken electronegativity that involves the second ionization potential *IP*_2_ of the dopant atom and their first *EA*^20^. The very good predictive power of this function which is based on textbook data, allowed us to predict trends in adsorption energy of O-atoms using quantities solely intrinsic to the dopant atoms at the surface. The systematic analysis of the electronic structure of the surface-dopant-O-atom complex revealed how the bands and newly formed electronic states sequentially follow a trend that depends on *IP* and *EA* and explains why these quantities could be used for devising predictive descriptors of adsorption^20^. The success of this descriptor of adsorption is because it includes quantities dependent on filled or valence states, the *IP*_*2*_, and on empty states, the *EA*. This makes the descriptor able to account for donation and back donation upon formation of chemical bonds at the surface. Because for adsorption of the same adsorbate onto different surfaces, the reference electron energy of the adsorbate is the same, the descriptor will grasp the relative differences between filled and empty states for different surfaces or different materials.

In this work we tested the usage of descriptors for predicting trends in adsorption of H-atoms on a series of transition metals of technological relevance that have fcc structure. We show that the first-stage electronegativity and the modified second-stage electronegativity of the metals are good descriptors of trends in adsorption at (111), (110) and (100) surfaces of fcc metals, and that these descriptors can be used in tasks of rational design or materials selection. The first-stage electronegativity of the metals correlates linearly with experimentally measured exchange current density, avoiding the volcano-like plot. These results also highlight the importance of averaging over different adsorbate coverages and over different surface planes for the modelling data to be closer to the experimental conditions in engineered catalytic reactor systems.

## Methods

### Literature data

Methodologies for literature data collection on hydrogen adsorption are detailed in the Supplementary Information to this paper.

### Computational details

**Fig. 1:**
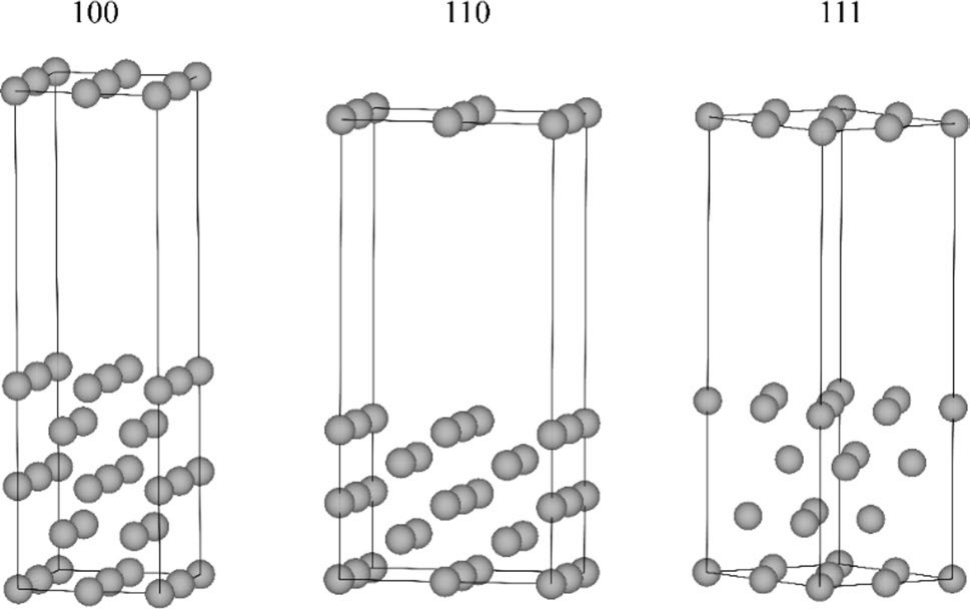
Supercell models with *p* (2 × 2) symmetry used to study adsorption of H-atoms onto the fcc transition metals: Ag, Au, Co, Cu, Ir, Ni, Pd, Pt, Rh.

Density functional theory (DFT) calculations were performed using the Vienna ab initio simulation package (VASP 5.4.1)^21^ with the Perdew–Burke–Ernzerhof (refs ^22,23^.) (PBE) exchange–correlation functional with ultrasoft pseudopotentials of the projector augmented wave^24,25^ (PAW) type. The PBE functional has shown good accuracy for describing the adsorption of H-atoms onto metal surfaces^26–28^, and also for surface and bulk properties of metals^29,30^. We tested the inclusion of descriptors of van der Waals interactions which are important in molecular adsorption, using the D3 correction^31^ as PBE-D3. However, the adsorption energies of H-atoms at the surfaces of these transition metals computed at the PBE level are orders of magnitude larger than the D3 correction to those energies which results in negligible relative differences— < 0.002 eV on average—in the adsorption energies obtained with and without the D3 correction. Because of this and to facilitate direct comparison with the literature, the data here presented has been computed at the PBE level. The energies herein reported are electronic energies at 0 K which allow accurate direct comparisons between preferred binding sites with similar chemical environments^32–34^. The (100), (110) and (111) fcc surfaces were modelled with *p*(2 × 2) surfaces using the supercell models shown in Fig. 1.

The supercells with periodic boundary conditions have a thickness of five atomic layers for (100) and (110) along the axis perpendicular to the surface planes and four atomic layers for (111), all with vacuum layers of 15 Å. These models have been employed in our previous works for the study of adsorption of H-atoms and are sufficient to reduce spurious interactions between the periodically repeating images^27,28,35^. Adsorption of H-atoms was investigated at one of the sides of the slabs. During optimization of the geometries, for (100) and (110), the three bottom layers were kept fixed while the remaining top layers as well as the H-atoms were allowed to relax. For (111) the two bottom layers were kept fixed while the remaining top layers and the H-atoms were allowed to relax. For geometry optimizations a plane wave cutoff of 500 eV and a Γ centered k-point mesh of (6 × 6 × 1) in the Monkhorst–Pack sampling scheme were used with a Gaussian smearing of 0.1 eV width. The self-consistent field (SCF) electronic energies were considered as converged when the change was smaller than 1 × 10^−5^ eV between cycles and the force acting on each of the atoms smaller than 0.002 eV Å^−1^. The study of the adsorption of H-atoms started with a single H-atom per supercell (0.25 ML). At this stage we searched for adsorption site preference. Subsequently the coverage was increased sequentially until one monolayer (1 ML). The definition of ML here used is that recommended by IUPAC^36^.

The adsorption energies of the H-atoms were determined as

1 $$\Delta E_{{{\text{ads}}}} H = E_{{(n{\text{H - }}M{\text{\_slab)}}}} - \left( {nE_{H} + E_{{M\_{\text{slab}}}} } \right)$$

where *E*_(*n*H-*M*_slab)_ is the energy of *n* H-atoms bound to a specific surface of a metal *M*; *E*_H_ is the energy of an H-atom in vacuum and *E*_*M*_slab_ is the energy of the supercell containing the bare surface of *M*. A more negative value for Δ*E*_ads_H implies stronger adsorption. The values of Δ*E*_ads_H are normalized per H-atom considering the following correspondence between ML and the number of adsorbed H-atoms per supercell: 0.25 ML = 1H; 0.5 ML = 2H: 0.75 ML = 3H and 1 ML = 4H. Two different average quantities have been devised to express the Δ*E*_ads_H. The average over the coverage for each surface plane for each metal is defined as

2 $$\Delta \overline{E}_{ads} H_{{\left( {100} \right)}} , \Delta \overline{E}_{ads} H_{{\left( {110} \right)}} , \Delta \overline{E}_{ads} H_{{\left( {111} \right)}} = \left( {\frac{{\Delta E_{ads} H_{{\left( {0.25} \right)}} + \Delta E_{ads} H_{{\left( {0.5} \right)}} + \Delta E_{ads} H_{{\left( {0.75} \right)}} + \Delta E_{ads} H_{\left( 1 \right)} }}{4}} \right)_{{\left( {100} \right), \left( {110} \right),\left( {111} \right)}}$$

where Δ*Ē*_ads_H_(100)_, Δ*Ē*_ads_H_(110)_, Δ*Ē*_ads_H_(111)_ are the average adsorption energies for the H-atom for each of the surfaces for a given metal; and Δ*E*_ads_H_(0.25)_, Δ*E*_ads_H_(0.5)_, Δ*E*_ads_H_(0.75)_, and Δ*E*_ads_H_(1)_ are the corresponding adsorption energies of the H-atom for each surface coverage—0.25 to 1 ML—expressed per H-atom. Because the differences in the surface energies for the three low Miller index surfaces of fcc metals are in general not large^37^, in a polycrystal all these surfaces can be present, even if in different extents, but will thus be responsible for the overall catalytic activity of the material. Hence, the average adsorption energy of the H-atoms for each metal here studied (Δ*Ē*_ads_H) was obtained as

3 $${\Delta }\overline{E}_{ads} H = \left( {\frac{{{\Delta }\overline{E}_{ads} H_{{\left( {100} \right)}} + {\Delta }\overline{E}_{ads} H_{{\left( {110} \right)}} + {\Delta }\overline{E}_{ads} H_{{\left( {111} \right)}} }}{3}} \right)$$

The electronic energy dependent chemical potential *µ*_*i*_ is defined as a function of ML fraction up to 1 ML as

4 $${\upmu }_{i}= {\left(\frac{\partial {\Delta E}_{ads}}{\partial {ML}_{i}}\right)}_{\left(100\right),\left(110\right),(111)}$$

where Δ*E*_ads_ is the adsorption energy of *i* H atoms. This expression does not include the zero-point vibrational energies because in previous works we found that this quantity does not contribute to changes in the trends of adsorption nor absorption for similar geometries^28,38^. The expression does not include also corrections for pressure nor temperature. The values of *µ* were thus determined from plots of Δ*E*_ads_H_*i*_* vs* ML for each surface leading to the quantities *µ*_*i*(100)_,* µ*_*i*(110)_ and* µ*_*i*(111)_ which are the coverage dependent chemical potentials for H-atom adsorption at the corresponding surfaces of each metal. Because each 0.25 increase in ML corresponds to an H-atom, the *µ*_*i*_ expressed per ML can be easily expressed per number of H-atoms by computing *µ*_*i*_/4. The average chemical potential *µ*_*avg*_ for adsorption of H at the surface of each metal here considered is determined as

5 $${\upmu }_{avg}= \left(\frac{{\mu }_{i(100)}+{\mu }_{i\left(110\right)}+{\mu }_{i(111)} }{3}\right)$$

## Results and discussion

### Analysis of literature data

It is thus expectable that, if for a given material the electronic properties of each surface, for example (100), (110) and (111), change in a consistent way and can be linked to certain trends in adsorption, the intrinsic properties of the materials such as *IP*, *EA*, and *χ* can also be related to the type of surface by a function. Correlations between surface plane dependent work function *W*, for the (100), (110) and (111) surfaces and bulk *IP* and *EA* that are independent of the surface are shown in the Supplementary Information Figure SI2 for a series of transition metals with fcc structure. The correlations between* W* and *IP*, *EA* and *χ* are not linear as expected because the electronic states of the surfaces that influence *W* depend on complex quantum mechanical effects that do not correlate linearly with *IP*, *EA* and *χ*. However, the fact that the data of Figure SI2 varies for the different surface planes in a tendentially linear fashion suggests that it is possible to correlate certain aspects of the performance of the surfaces with bulk properties of the materials if we obtain an adequate parameter that “adapts” the bulk properties *χ*, *EA* and *IP*, to the specific surface plane, taking into account a specific surface process, that itself it is surface plane dependent. Such simplification that does not depend on descriptors that need complex computational data would allow a fast track for evaluation of performance and for the design of materials and systems that are aimed at specific surface performance.

### Coverage dependent adsorption energies

Adsorbate coverage is an important parameter that determines the adsorption energy^39,40^. We have previously demonstrated that even for defective surfaces, adsorbate coverage has an important role both in the formation and in the magnitude of dipoles and in the strength of adsorption^17,41,42^. Long-range adsorbate–adsorbate effects have been determined to amount to 15 Å in Cu for coverages as low as 1/16 ML and 1/32 ML. For S-atoms, these interactions can affect the magnitude of the adsorption energy by 0.4 eV and the magnitude and sign of local dipole momenta—from + 1.217 D for adsorption at neighboring sites, to − 2.390 D for adsorption at a distance of 18 Å^17^. For applications in high performance catalytic reactors however, higher coverages closer to 1 ML have to be considered. We started by studying the coverage dependence of Δ*E*_ads_H from 0.25 to 1 ML. The resulting data is shown in Supplementary Information Figure SI3 and the obtained chemical potentials *µ*_*i*_ are summarized in Table 1.

**Table 1:** Electronic energy dependent chemical potentials *µ*_*i*_ expressed as coverage of H-atoms at the corresponding surfaces from 0.25 to 1 ML. *µ*_*i*_ is defined in Eq. (4).

	*µ*_*i*_ (ev)/ML			*b* (eV)		
(hkl)	100	110	111	100	110	111
Ag	− 8.166	− 8.856	− 4.230	0.210	0.588	− 0.604
Au	− 9.011	− 7.534	− 6.392	0.442	− 0.123	− 0.339
Co	− 11.199	− 10.613	− 4.615	0.114	− 0.581	− 1.726
Cu	− 9.325	− 10.004	− 4.749	0.009	0.553	− 1.381
Ir	− 11.016	− 10.667	− 10.464	− 0.125	− 0.076	− 0.068
Ni	− 12.618	− 11.337	− 7.837	1.215	0.506	− 0.309
Pd	− 11.384	− 8.571	− 8.722	0.968	− 0.089	− 0.142
Pt	− 10.559	− 11.131	− 10.282	− 0.163	− 0.093	− 0.139
Rh	− 11.142	− 11.511	− 9.388	0.554	0.827	− 0.121

**Fig. 2:**
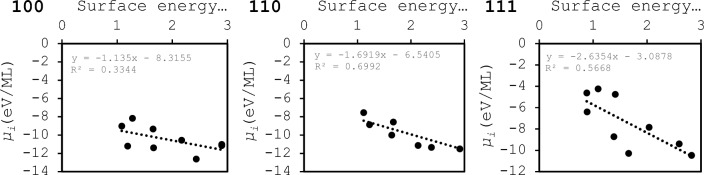
*µ*_*i*_ (eV/ML) as a function of the surface energies (eV) for the series of fcc metals here studied. The surface energy of Co(110) is non-existent in the literature and has not been included.

The dependence of Δ*E*_ads_ with ML is linear for all metals and surface planes except for Cu(111) and Co(111) most likely due to spin effects as these surfaces are known to undergo adsorbate induced transitions that can lead to non-zero spin momenta and to localized dipoles upon adsorption^17,43–45^. The extent of surface reconstruction for the same surface planes at the same coverage of H-atoms is different for different metals as shown in Figure SI4 for Ag and Co. This highlights the importance of studying both different coverages and also allowing the models to relax during geometry optimization to find the real minima. The chemical potentials expressed as a function of surface energy reveal how the process of adsorption resembles that of reconstructing the bulk material: chemical bonds between a surface and an adsorbate which are more similar to the bonds in the bulk metal lead to stronger correlations with the surface energy. The correlations between *µ*_*i*_ and the surface energies of the fcc metals are shown in Fig. 2.

There is a strong dependency of *µ*_*i*_ with the surface energy for the sets of surfaces (110) and (111), while for the set (100) the correlation is weak. The 110 surface is the more open of the three which leads to more degrees of freedom for bonding with H-atoms in terms of their disposition ie. top, bridge hollow etc., as highlighted in Figure SI4. The effect of the changes in binding site preference reflects in the correlation between surface coverage and *µ*_*i*_. However, the 110 surface is not the surface for which the binding site preference changes the most with coverage. For the 100 set of surfaces, the changes in adsorption site preference as the coverage of H-atoms changes are the most extreme from the complete set of metals here studied. These effects can be attributed to adsorbate–adsorbate effects that change both magnitude and direction of local dipole momenta at different coverages^17^. These results show the importance of allowing adsorbate geometry relaxation in computations of adsorption dependent such quantities. The consequences of the more extensive changes in binding site preference as a function of coverage (100), are different correlations between the adsorption energy and the descriptors of adsorption that we are devising in this work as it will be shown in the next section.

### Descriptors of adsorption energy

**Fig. 3:**
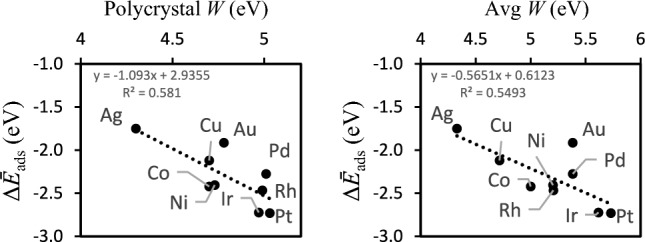
Left panel: experimentally measured work functions *W* for polycrystals of the metals^50^ and their correlation with the average adsorption energies of the H-atom Δ*Ē*_ads_H (eV) obtained according to Eq. (3), over coverage and surface planes. Average (avg) *W* obtained by averaging the *W* of the (100), (110) and (111) surfaces retrieved from literature^51^.

Simple descriptors of Δ*E*_ads_ that predict relative differences in this quantity in catalytic reactors could boost the design of catalysts^46–48^. Predictors of relative Δ*E*_ads_H can thus help design better electrodes for electrolysis and fuel cells among other systems where Δ*E*_ads_H is of relevance^46,49^. In this context, we tested how well our averaging methodology can grasp the non-linearities observed in experiments by correlating experimentally measured *Ws* of the metals and our average adsorption energies of the H-atom Δ*Ē*_ads_H (eV) obtained according to Eq. (3). The resulting data is shown in Fig. 3. The data shows that our averaging strategy grasps experimental conditions better than other modelling approaches that focus on single coverages as the linear correlations show.

Additionally, the correlation between Δ*Ē*_ads_H and the two sets of experimental *W* shows that averaging adsorption energies over coverage and over the surfaces with lower surface energies is a good approach to correlate intrinsic physical properties with adsorption performance. This is because in the polycrystals the three surfaces (100), (110) and (111) can occur because the differences in their energies are small^37^. Averaging over data obtained for each surface is thus a better approximation for polycrystals. The linear correlations between Δ*E*_ads_H at each coverage for each type of surface and the *W* of each surface are weak—0.11 < R^2^ < 0.48—for most of the surfaces and coverages as the collection of data in Supplementary Information Figure SI5 shows. The exceptions are the higher coverages 0.75 and 1 ML at the (111) surfaces and the 1 ML coverage at the (110) surfaces. This shows that *W* cannot account for the shifts in adsorbate binding site preference that occur when the coverage increases, as we have already discussed above. The set of (100) surfaces are those where these effects are more significant because of the larger number of possible adsorbate binding sites. This demonstrates that geometric and electronic effects go hand in hand and methods for prediction of adsorption must account for these. Averaging over both coverage and surface planes, and finding the energy minima for each geometry at each coverage accounts substantially for both effects as Fig. 3 demonstrates.

### Descriptors of catalytic activity

The exchange current density, *i*_*0*_, in electrochemical reactions is analogous to the rate constant in chemical reactions. For a homologous series of reactions, Brønsted, Evans and Polanyi (BEP) have shown that there is a linear correlation between the transition state energies—that in turn affect the corresponding reaction rate constants—and the adsorption energies in what is now known as the BEP relation^52,53^. Several authors have explored this correlation for the successful prediction of catalytic performance of metal surfaces^54^, including ourselves, in our case for surfaces of oxides in solution^12^. Here we explore the correlation between *i*_*0*_ at the hydrogen evolution electrode (HEE) and the Δ*E*_ads_H with an approach fundamentally similar to what was tested previously, but with important differences^55^. The difficulties that the authors of reference^55^ encountered are due to the usage of data obtained with different methods for the determination of Δ*E*_ads_H which in turn have different errors. Additionally, the study considered only ¼ ML and 1 ML. In the present study we consider the overall average of the Δ*E*_ads_H, defined as Δ*Ē*_ads_H that in turn is the average of the Δ*E*_ads_H obtained for each surface plane also averaging over the different coverages as described in Eq. (3). This makes the quantity Δ*Ē*_ads_H grasp both surface coverage effects on the Δ*E*_ads_H and the effects of the surface index in this quantity.

**Fig. 4:**
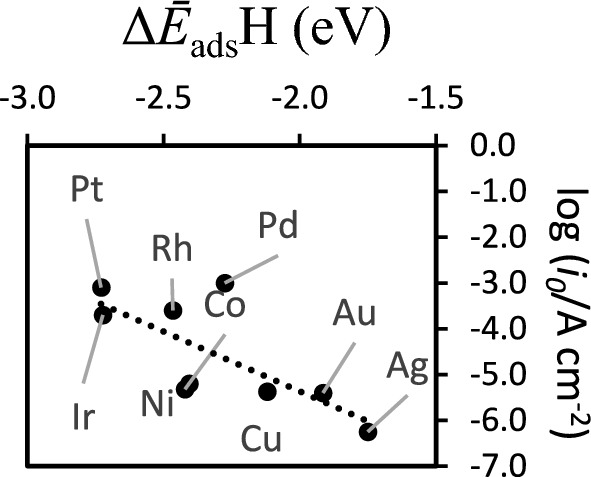
Dependency of the exchange current density at the hydrogen evolution electrode log (*i*_*0*_/A cm^−2^) with the adsorption energy of the H-atom Δ*Ē*_ads_H (eV) obtained according to Eq. (3). The linear fit has a slope *m* =  − 2.6, an intercept at origin *b* =  − 10.59 and a goodness of fit of 0.60.

In Fig. 4, the more reactive metals are at the left of the plot while the less reactive and unreactive are at the right as previously observed for a volcano plot of the activities of these metals^55^. However, the close to linear dependency of *i*_*0*_ with Δ*Ē*_ads_H can be attributed to the fact that as observed for the *Ws* data of Fig. 3, the averaging of the adsorption energies in terms of surface planes and in terms of surface coverages grasps the experimental conditions to a better extent that if only selected surface planes or coverages are used. This is because of the highly dynamical nature of the reactions of evolution of hydrogen whose rate of formation depends on the Δ*E*_ads_H but the rate of desorption from the surface depends on the surface coverage as well on the relation between Δ*E*_ads_H and surface coverage, that we express here as the surface coverage dependent chemical potential, *µ*_*i*_. We have previously succeed in using a similar averaging approach for the prediction of the formation of H_2_(g) at the surface of Cu(110), where averaging over different adsorbate conformation energies and coverages was necessary for accounting for the contributions of adsorbate dispositions that can occur in the real systems because these have similar energies^35,41,42,56^.

**Fig. 5:**
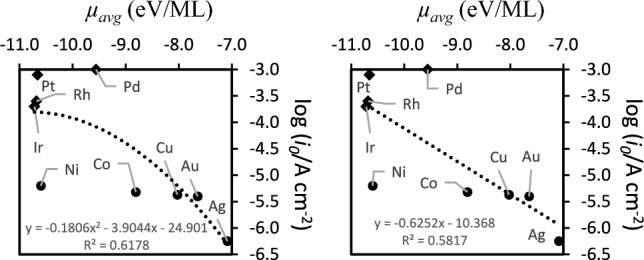
Average chemical potential for H-atom adsorption (*µ*_*avg*_) and correlation with the exchange current density at the hydrogen evolution electrode (*i*_*0*_). *µ*_*avg*_ were determined from Eq. (5). Experimental values of *i*_*0*_ retrieved from^55^ and references therein. Less active catalysts (circles), more reactive catalysts (diamonds).

The quadratic correlation between *µ*_*avg*_ and *i*_*0*_ in Fig. 5 shows that a volcano-like trend of quadratic nature may apply to these two quantities. Fitting the data with linear functions leads to very poor correlations for elements whose *µ*_*avg*_ are more negative than − 9 eV/ML and a quadratic function leads to better goodness of fit. The data clearly shows two groups of materials in terms of correlation between *µ*_*avg*_ and *i*_*0*_. It shows also that the driving force for adsorption reaches a plateau in terms of its effect in the electrode performance.

**Fig. 6:**
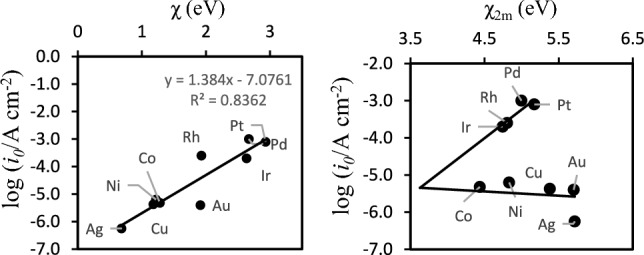
First-stage electronegativity χ (eV) and modified second-stage electronegativity χ_2m_ (eV) determined according to Eqs. (6) and (7) and their correlations with the exchange current density *i*_*0*_. χ and χ_2m_ are obtained from the well-known quantities IP and EA. Experimental values of *i*_*0*_ retrieved from^55^ and references therein.

We previously used primary quantities for the prediction of oxygen adsorption and dissociation on pure and doped Al surfaces^20^ and in the description of catalytic activity of oxides in solution^12^, correlating computational with experimental data. The first of those quantities that we will test here is the first-stage electronegativity (χ) as defined by Mulliken. The second quantity is what we called “modified second-stage electronegativity” where instead of the quantities used for the second-stage electronegativity in the definition by Mulliken, we employed the second ionization potential IP_2_ of the metal and the one electron affinity EA. The details are given elsewhere^12,20^. Both expressions are

6 $$\upchi =\frac{IP+EA}{2}$$

7 $${\upchi }_{2\text{m}}=\frac{{IP}_{2}+EA}{4}$$

where χ_2m_ is the modified second-stage electronegativity, IP is the first ionization potential of the metal, IP_2_ is the second ionization potential of the metal—surface independent quantities–and EA is the electron affinity of the metal—a surface independent quantity as well. IP, IP_2_ and EA are quantities—for the elements—retrieved from common databases. The details of the choice of IP_2_ instead of IP (first ionization energies) for devising χ_2m_ are given in our original work. In general, IP_2_ together with EA accounts for the different extents of donation-backdonation that occurs when the H-atom binds to the surface^57–61^, and the relaxation of the remaining orbitals in the d-band of the metal as described in the Newns-Anderson model. For a given adsorbate, depending on the metal, the balance between donation-backdonation of electron density will change. Our descriptor (χ_2m_) grasps the correct phenomenology of these processes for dopants on Al surfaces and their effect on oxygen adsorption^20^. For the set of fcc metals here study, the plots of the exchange current density (*i*_*0*_) in a hydrogen evolution electrode versus χ and χ_2m_ are shown in Fig. 6.

The data of Fig. 6 shows an excellent correlation between the first-stage electronegativity of the metals (χ) and *i*_*0*_. This shows that χ can in fact be used as a linear descriptor in the search for catalysts for HEE. Further support to this observation is also given by χ_2m_ in Fig. 6. The “sideways volcano plot” resultant from correlating χ_2m_ with *i*_*0*_ provides considerable insight into the phenomenological description of bonding: donation–backdonation, between the H-atom and the surfaces as it will be discussed in the next section.

### Theoretical and conceptual framework of the descriptors of catalytic activity

In the Newns–Andersson model the energetic positioning of the d-band center with respect to the orbitals of the adsorbate explains certain trends in catalytic activity. The direct correlation between the model and a surface process is with regard to adsorption energies, that in turn relate with frequency of events or with rates by the BEP relation leading to an indirect correlation between the d-band derived quantities and the frequency or rates of events. Bonding between an adsorbate to a series of surfaces can thus be rationalized in terms of how high or low the d-band center and/or edge for the different materials lay with respect to one another. However, this model does not account directly for the role of electrons of the metal that lay in other orbitals such as p and s and nor accounts for effects of Pauli exclusion for example. The relationship between the energies of the d-band and the p and s bands of the metal is considered only indirectly because certain shifts in the d-band can lead to shifts in the p and s energies, but this is not a universal rule, it is in fact far from it^16,62,63^. The details of this model and how the chemical bonding between different surfaces and adsorbates come into play have been extensively described in reference texts^62,64,65^.

In our model here presented we use quantities that despite their simplicity contain the necessary information for the description of donation and backdonation from and to adsorbates. Many of the data here computed is correlated with polycrystalline and “poly-Miller-index” surfaces. The averaging over coverage and surface plane that we have employed is necessary to obtain such correlations which in turn show that there are primary electronic properties of the materials which are surface plane independent and still determinant for the description of the activity of the metal as a HEE. However, such primary quantities require an algebraic workout to obtain a descriptor that contains the correct assessment of electron donation and backdonation to and from the adsorbate, and that weighs these two processes of donation and backdonation. The typical quantities devised for example in conceptual DFT^66^, are related with where the electron orbitals are located energetically. As we previously employed for predicting reactivity of transition metal and actinide oxides^12^, this allows a description of hardness, softness and similar properties that depend on the work necessary to attach and detach electrons. However, the driving forces for the loss or gain of electrons are equally well described by the IP, IP_2_ and EA provided the correct algebraic expression is used.

**Fig. 7:**
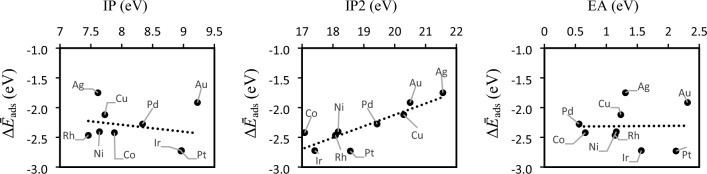
First ionization potential IP, second ionization potential IP_2_ and electron affinity EA and their correlation with the average adsorption energies Δ*Ē*_ads_H of the H-atom to the corresponding metals obtained according to Eq. 3. The values of IP, IP_2_ and EA used here are retrieved from common databases for the elements and are surface independent properties.

The plots correlating the primary and secondary quantities used to determine the first-stage and second-stage electronegativities, χ and χ_2m_ respectively, and the average adsorption energies of H-atoms, Δ*Ē*_ads_H are given Fig. 7.

The first ionization potential IP accounts for the energy of the highest occupied electronic states of the metal, with no distinction between orbitals, d or p. IP accounts for the energy—or work necessary if we wish to express so—for the electron transfer from the metal to occur. The electron affinity EA accounts for the ability of the metal to accommodate electrons. This quantity can be positive or negative but for metals it is mostly positive, meaning that energy is released when the metal atom accepts an incoming electron. The second ionization potential, IP_2_ accounts for the energy necessary to remove a second electron from the metal. This quantity includes thus the relaxation of the metal orbitals after the first electron has been removed which in turn account for electronic relaxation effects in the donation–backdonation to and from the adsorbate. IP and IP_2_ are largely related but include different physical descriptors of the electronic structure and this is demonstrated by the different degrees of correlation and signs of the slopes of their plots versus Δ*Ē*_ads_H in Fig. 7. The first-stage electronegativity χ that contains IP and EA will thus account for the balance between the energy gained and lost in the donation-backdonation to and from the H-atom to the surfaces. The second-stage electronegativity χ_2m_ will account for relaxation effects of the orbitals of the metal in the bonding with the adsorbate. This is because it accounts for the ease of removal of a second electron from the metal, which in turn is a quantity that contains information about the ease of relaxation of the highest energy electronic bands of the metal. Interestingly, the quantities IP and EA, despite leading to poor correlations with the adsorption energy of the H-atoms as shown in Fig. 7, are included both in the linear correlation between χ and *i*_*0*_ and the volcano like correlation between χ_2m_ and *i*_*0*_ shown in Fig. 6 that result in strong correlations. This shows that χ and χ_2m_ contain the essential information of electronic structure features of the metals that determine the dynamical factors that result in different *i*_*0*_ in the HEE. χ and χ_2m_ have thus the potential to be used as descriptors in the search for HEE materials and other electrode or catalytic materials where adsorption of H-atoms is a key process. The simplicity of these functions means that they can be easily implemented in machine learning algorithms for catalyst screening and design.

## Conclusions

The search for a large set of literature data on H-atom adsorption on fcc metals shows that data computed by different authors with different methods have uncertainties that do not allow the creation of statistically significant sets of data for usage in analysis of properties with good accuracy. Thus we present here a large set of DFT data on H-atom adsorption on the surfaces of fcc metals that is both coverage and surface plane dependent. Despite our set of data being obtained for discrete surface planes and coverages, our averaging procedure leads to data that can be correlated with experimental properties obtained for polycrystals. This shows that our averaging procedure can approximate the DFT data to the diversity of coverages and surfaces that experiments deal with. The correlations between surface energies, work functions and adsorption related properties if obtained for single surface planes and coverages without averaging are poor. This is because changing coverage implies changes in adsorbate biding site preferences for certain planes also accompanied by different extents of surface reconstruction. Our averaging methodology grasps these effects and allows the correlation between H-atom adsorption and experimental data obtained for polycrystals as shown by the linear correlations that we obtained between DFT data and exchange current density (*i*_*0*_) in a hydrogen evolution electrode.

We also propose descriptors of electrode activity using the first-stage (χ) and second-stage electronegativity (χ_2m_). Because these quantities depend on electron energies of the metals, they include the essential information that determines donation and backdonation to and from adsorbates and even effects of electronic relaxation. The result is that can χ be linearly correlated with *i*_*0*_ and χ_2m_ can be correlated with *i*_*0*_ via a function that resembles the typical volcano plot. Because of these strong correlations, especially between *i*_*0*_ and χ with is strongly linear, both χ and χ_2m_ can be used as robust descriptors of catalytic activity in search and in the design of new materials for usage as hydrogen evolution electrodes. The direct dependence of χ and χ_2m_ on electron energy levels simplifies considerably the number of degrees of freedom to consider in the design of catalysts.

## Supplementary Information

Supplementary Information.

## Data Availability

The data generated is available from the corresponding author upon reasonable request.
