# Supplementary material for: Hydrogen adsorption on fcc metal surfaces towards the rational design of electrode materials
Source: Sci Rep. 2024 Sep 9;14:20972. doi: 10.1038/s41598-024-71703-w (PMC11385180; doi:10.1038/s41598-024-71703-w)
Supplement: Supplementary file 1 — Supplementary Information. [file 41598_2024_71703_MOESM1_ESM.pdf]

Supplementary information for

Hydrogen adsorption on fcc metal surfaces towards the rational design of electrode materials

Cláudio M. Lousada* and Atharva Kotasthane

Department of Materials Science and Engineering, KTH Royal Institute of Technology, SE-100 44 Stockholm, Sweden

*correspondence: cmlp@kth.se

2. Supplementary Information for: Methods

2.1. Literature Data

Computational literature data on hydrogen adsorption shows that the typical coverages studied are between 0.25 ML to 1ML and the most common software are VASP and Quantum Espresso with various functionals and pseudopotentials. Only the (100), (110), and (111) surfaces are considered in this discussion because these have the lowest surface energies and thus occur most frequently in the real materials. Many authors report the H-atom adsorption data with respect to diatomic hydrogen as the reference state. This was corrected using the H_2(g) bond dissociation energy obtained from the corresponding DFT functional used in the corresponding work, to make the necessary comparisons and the data assessment possible.[1, 2]

For silver, the (100) and (111) have been the most studied surface planes and only one reference is available for (110). [3-8] Aluminum data is primarily for the most stable surface (111) and only one work focused on hydrogen adsorption on Al(100).[9] Both modelling and experimental data show that aluminum is not particularly efficient as H-atom adsorption material, but instead absorption to the bulk takes place [10-12]. Gold was also investigated by different authors using different methods which lead to data scatter [3, 4, 7, 13, 14]. Copper is a significant metal that has been widely investigated by many researchers concerning the (100), (110), and (111) surfaces. It is a common observation from all authors that hydrogen adsorbs well on Cu surfaces [3, 4, 11, 14-19]. Nickel has been thoroughly investigated at the (100), (110), and (111) planes. It is a well-studied element due to its high H-atom adsorption capacity which is reflected in the hydrogen adsorption energy [3, 7, 14, 20-23]. Hydrogen adsorption on the surfaces of palladium has been well documented and the data scatter is the smallest we found among all fcc metals [3, 7, 14, 24-26]. Platinum is one of the costliest metals, yet it leads to one of the strongest H-atom adsorption among all fcc metals making it the perfect metal for hydrogen fuel cell applications from this technical point of view. It also is the most studied H-atom adsorbent [3, 4, 7, 24, 27-29]. Rhenium is a particularly stable element at high temperatures, accounting for approximately 30% of the catalysts used by volume. It is an extremely costly metal because it is one of the rarest materials accessible, yet it is employed in catalysis. Experiments and modelling have demonstrated that it is second only to platinum in terms of hydrogen adsorption performance [7, 14, 30-33]. Fcc Co is an interesting material for energy applications [34, 35]. Thin films of fcc Co have been the goal of several recent studies [36, 37].

The exchange current density is an important parameter for the performance of systems that rely on electrodes [38]. The performance of electrodes depends on their composition and structure which have a significant impact on the exchange current density (i_0). We used (i_0) as a descriptor to test if the computed data for sets of perfect surfaces and different coverages with our averaging procedure can describe experimental data. The experimental data for exchange current density (i_0) for reactions of hydrogen evolution has been retrieved from the literature [39]. Because of significant differences between the values of two references, the i_0 for Ag was determined as the average between reference [40] and [41]. For the remaining metals, whenever multiple values exist, these have also been averaged.

3. Supplementary Information for: Results and Discussion

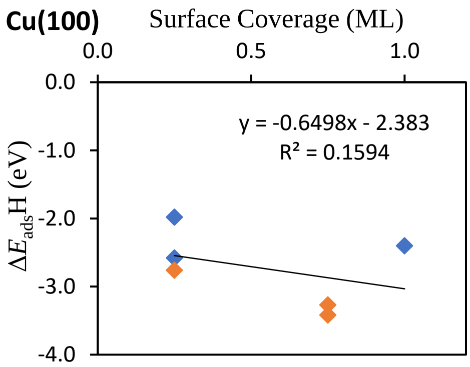

Figure SI1. ΔE_{ads}H (normalized per H-atom) on Cu(100) as a function of coverage (ML) after data from different authors. Orange is reference [4], Blue is reference [19].

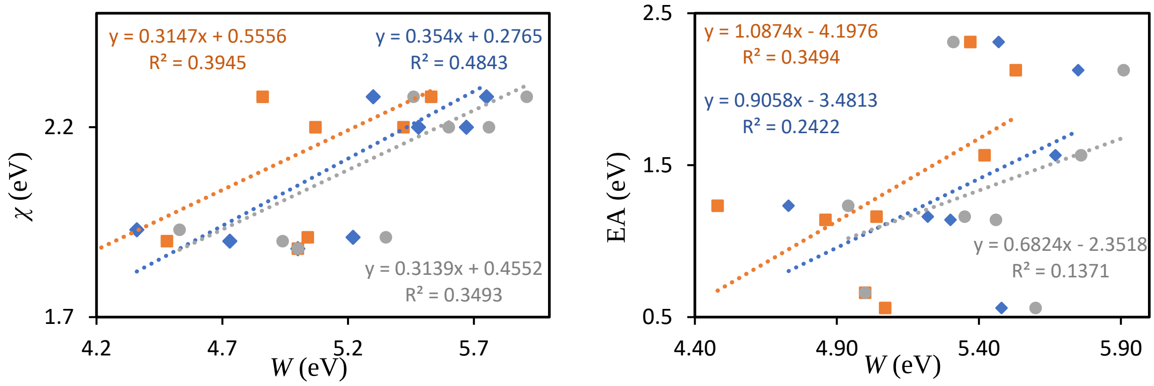

Figure SI2. Correlations between the surface independent first stage electronegativity (χ) and electron affinity (EA) and the surface dependent work function (W) for the transition metals with fcc structure: Ag, Au, Co, Cu, Ir, Ni, Pd, Pt, Rh. (100) blue diamonds, (110) orange squares, (111) grey circles.

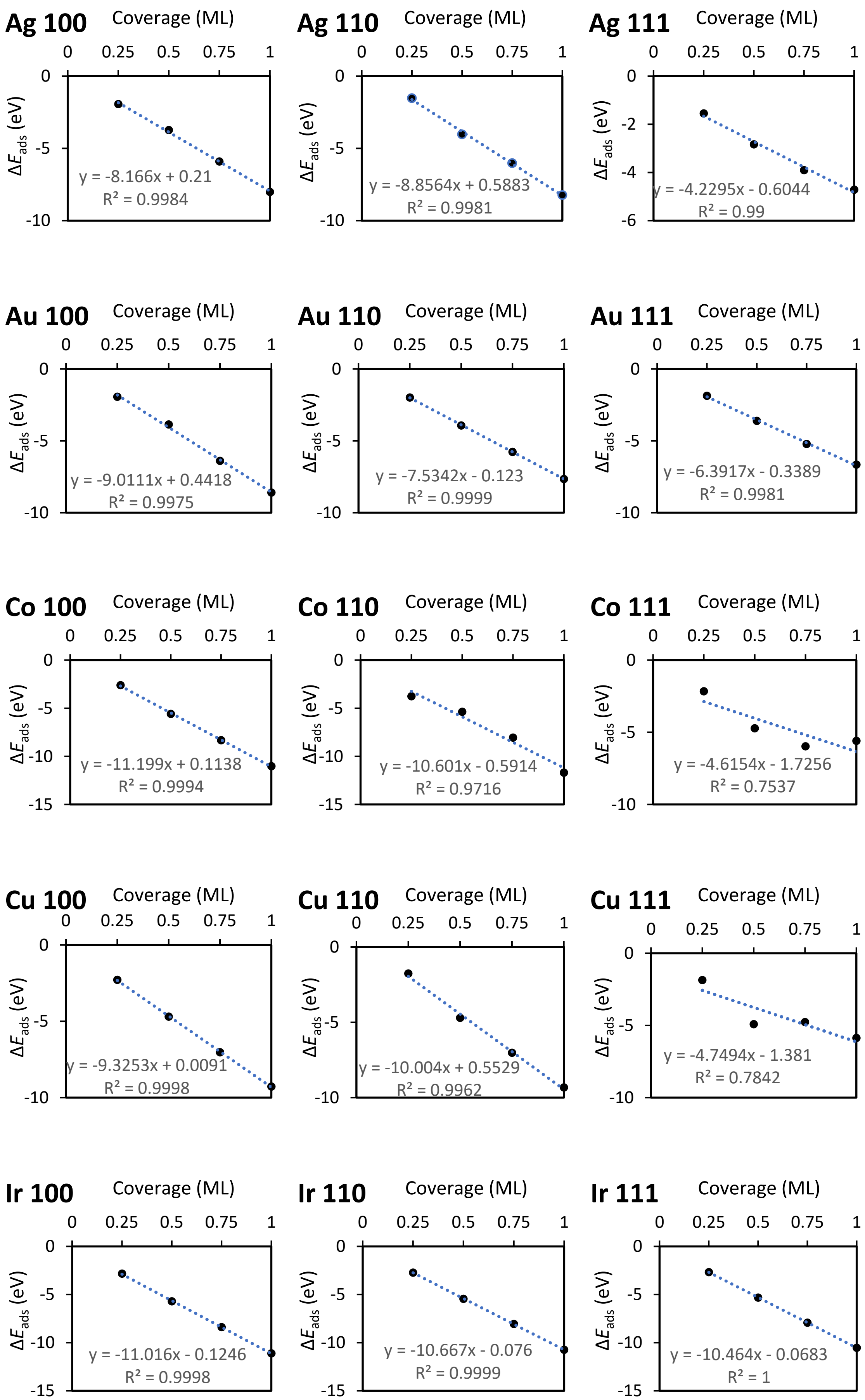
Ag 100
Coverage (ML)
ΔEads (eV)
y = -8.166x + 0.21
R² = 0.9984
Ag 110
Coverage (ML)
ΔEads (eV)
y = -8.8564x + 0.5883
R² = 0.9981
Ag 111
Coverage (ML)
ΔEads (eV)
y = -4.2295x - 0.6044
R² = 0.99
Au 100
Coverage (ML)
ΔEads (eV)
y = -9.0111x + 0.4418
R² = 0.9975
Au 110
Coverage (ML)
ΔEads (eV)
y = -7.5342x - 0.123
R² = 0.9999
Au 111
Coverage (ML)
ΔEads (eV)
y = -6.3917x - 0.3389
R² = 0.9981
Co 100
Coverage (ML)
ΔEads (eV)
y = -11.199x + 0.1138
R² = 0.9994
Co 110
Coverage (ML)
ΔEads (eV)
y = -10.601x - 0.5914
R² = 0.9716
Co 111
Coverage (ML)
ΔEads (eV)
y = -4.6154x - 1.7256
R² = 0.7537
Cu 100
Coverage (ML)
ΔEads (eV)
y = -9.3253x + 0.0091
R² = 0.9998
Cu 110
Coverage (ML)
ΔEads (eV)
y = -10.004x + 0.5529
R² = 0.9962
Cu 111
Coverage (ML)
ΔEads (eV)
y = -4.7494x - 1.381
R² = 0.7842
Ir 100
Coverage (ML)
ΔEads (eV)
y = -11.016x - 0.1246
R² = 0.9998
Ir 110
Coverage (ML)
ΔEads (eV)
y = -10.667x - 0.076
R² = 0.9999
Ir 111
Coverage (ML)
ΔEads (eV)
y = -10.464x - 0.0683
R² = 1

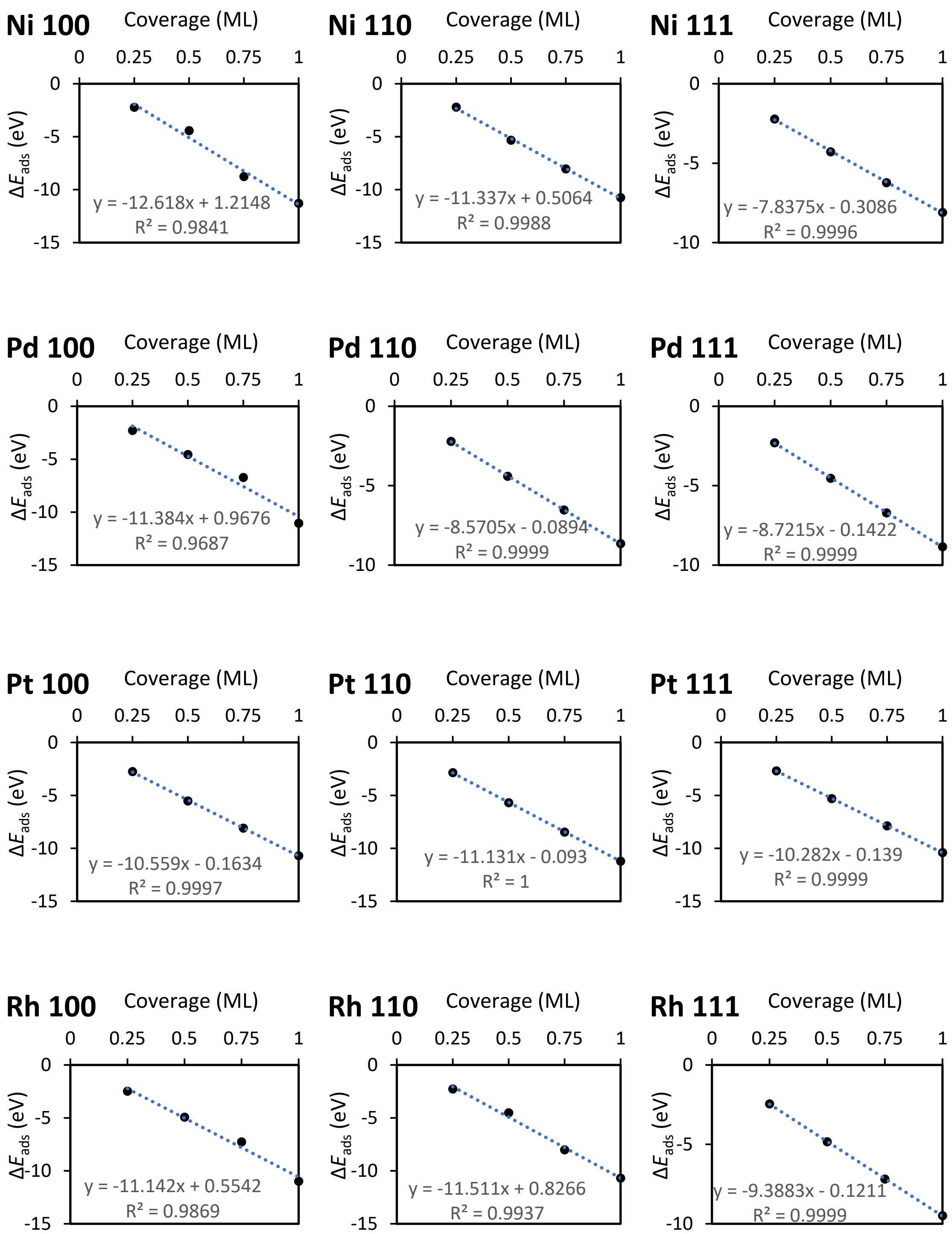

Figure SI3. Coverage dependent adsorption energies (ΔE_{ads}) of H-atoms. ΔE_{ads} is expressed as the total change in energy for the adsorption of the corresponding number of H-atoms for each coverage as expressed in equation 4. This data was used to obtain the chemical potentials μi for H-atom adsorption for each surface plane of each element.

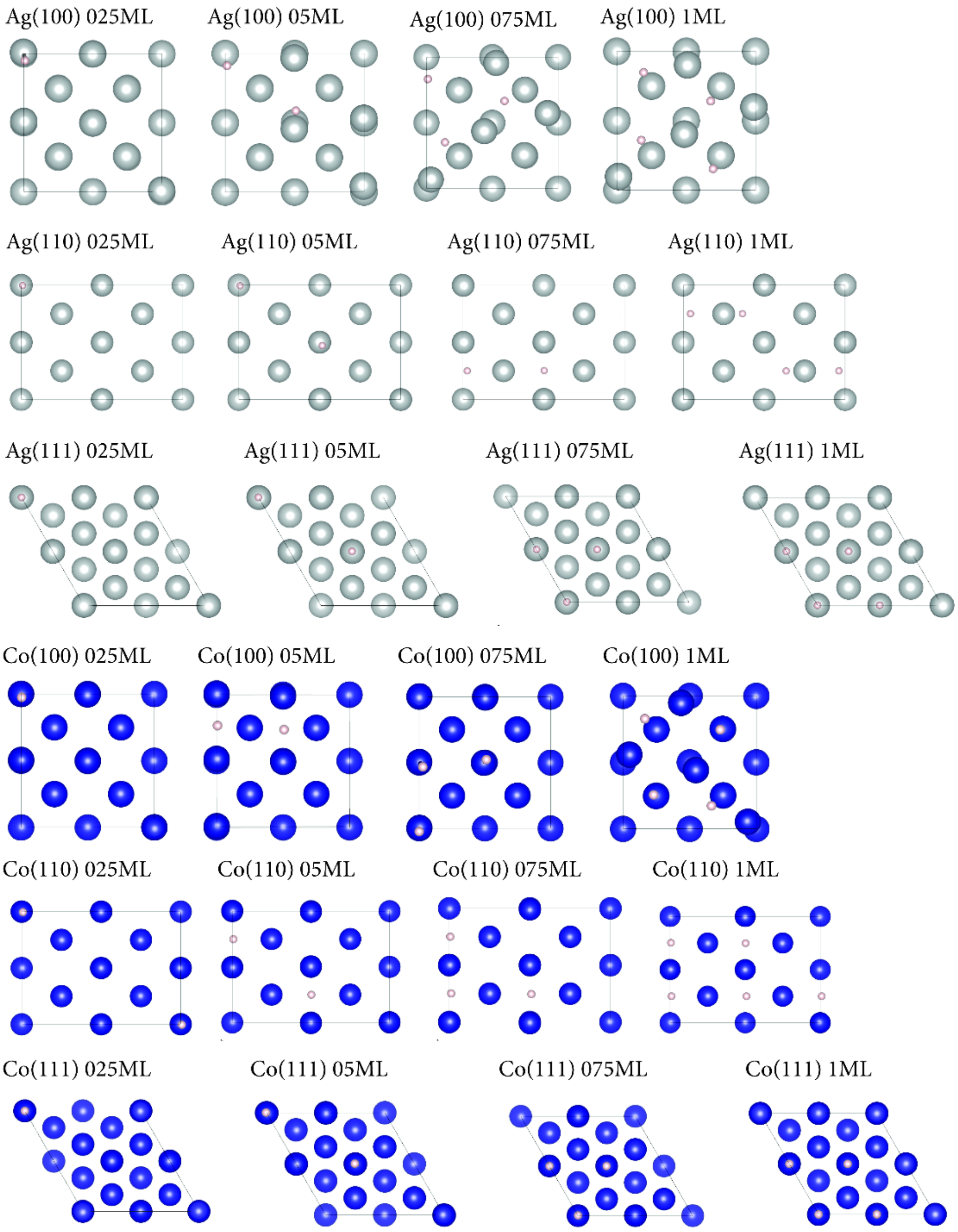

Figure SI4. Coverage dependent adsorption of H-atoms to Ag and Co surfaces. Note the different extents of surface reconstruction for the same surface planes and coverages between the two metals. Ag (grey), H (pink), Co (blue).

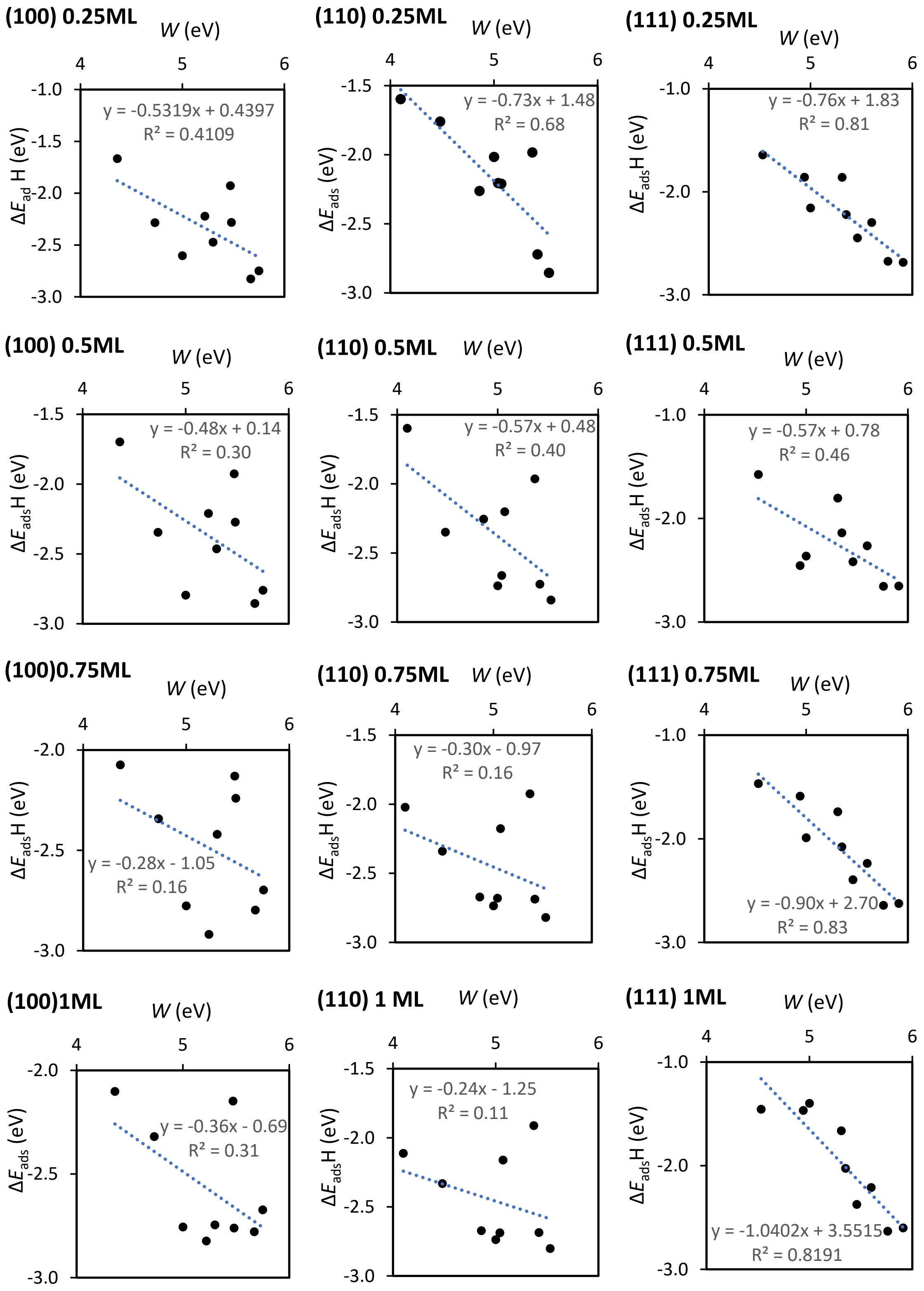

Figure SI5. Adsorption energies per H-atom ΔE_{ads}H (eV) for each surface plane (100), (110) and (111) and ML coverages 0.25, 0.5, 0.75 and 1 for the metals with fcc structure: Ag, Au, Co, Cu, Ir, Ni, Pd, Pt, Rh and their dependence on the work function (*W*) of each of the corresponding surfaces (100), (110) and (111) retrieved from literature [42].

3.1. Hydrogen adsorption data

Table SI1. Hydrogen atom adsorption data computed in this work. Monolayer coverage (ML); Number of H-atoms *n*H; adsorption energy for the *n* H-atoms (Δ*E* (eV)); adsorption energy per H-atom (ΔE (eV)/H-atom); adsorption energy per ML, for ML ≤ 1 (ΔE (eV) /ML).

**Ag(100)**						**Ag(110)**						**Ag(111)**				
ML	*n*H	ΔE (eV)	ΔE (eV) /H-atom	ΔE (eV) /ML		ML	*n*H	ΔE (eV)	ΔE (eV) /H-atom	ΔE (eV Ag) /ML		**ML**	*n*H	ΔE (eV)	ΔE (eV) /H-atom	ΔE (eV) /ML
0.25	1	-1.93	-1.93	-7.72		0.25	1	-1.52	-1.52	-6.07		0.25	1	-1.55	-1.55	-6.18
0.5	2	-3.73	-1.87	-7.46		0.5	2	-4.02	-2.01	-8.04		0.5	2	-2.83	-1.41	-5.66
0.75	3	-5.90	-1.97	-7.87		0.75	3	-6.02	-2.01	-8.03		0.75	3	-3.90	-1.30	-5.20
1	4	-8.01	-2.00	-8.01		1	4	-8.23	-2.06	-8.23		1	4	-4.71	-1.18	-4.71
**Au(100)**						**Au(110)**						**Au(111)**				
ML	*n*H	ΔE (eV)	ΔE (eV) /H-atom	ΔE (eV) /ML		ML	*n*H	ΔE (eV)	ΔE (eV) /H-atom	ΔE (eV) /ML		ML	*n*H	ΔE (eV)	ΔE (eV) /H-atom	ΔE (eV) /ML
0.25	1	-1.93	-1.93	-7.71		0.25	1	-1.98	-1.98	-7.93		0.25	1	-1.86	-1.86	-7.44
0.5	2	-3.85	-1.93	-7.71		0.5	2	-3.93	-1.96	-7.85		0.5	2	-3.61	-1.80	-7.22
0.75	3	-6.39	-2.13	-8.52		0.75	3	-5.77	-1.92	-7.69		0.75	3	-5.22	-1.74	-6.95
1	4	-8.59	-2.15	-8.59		1	4	-7.65	-1.91	-7.65		1	4	-6.65	-1.66	-6.65
**Co(100)**						**Co(110)**						**Co(111)**				
ML	*n*H	ΔE (eV)	ΔE (eV) /H-atom	ΔE (eV) /ML		ML	*n*H	ΔE (eV)	ΔE (eV) /H-atom	ΔE (eV) /ML		ML	*n*H	ΔE (eV)	ΔE (eV) /H-atom	ΔE (eV) /ML
0.25	1	-2.60	-2.60	-10.41		0.25	1	-3.76	-3.76	-15.02		0.25	1	-2.16	-2.16	-8.63
0.5	2	-5.59	-2.80	-11.18		0.5	2	-5.36	-2.68	-10.73		0.5	2	-4.73	-2.36	-9.45
0.75	3	-8.33	-2.78	-11.10		0.75	3	-8.06	-2.69	-10.74		0.75	3	-5.97	-1.99	-7.96
1	4	-11.02	-2.76	-11.02		1	4	-11.70	-2.92	-11.70		1	4	-5.59	-1.40	-5.59
**Cu(100)**						**Cu(110)**						**Cu(111)**				
ML	*n*H	ΔE (eV)	ΔE (eV) /H-atom	ΔE (eV) /ML		ML	*n*H	ΔE (eV)	ΔE (eV) /H-atom	ΔE (eV) /ML		ML	*n*H	ΔE (eV)	ΔE (eV) /H-atom	ΔE (eV) /ML
0.25	1	-2.28	-2.28	-9.14		0.25	1	-1.76	-1.76	-7.04		0.25	1	-1.86	-1.86	-7.43
0.5	2	-4.69	-2.34	-9.38		0.5	2	-4.70	-2.35	-9.40		0.5	2	-4.91	-2.46	-9.82
0.75	3	-7.03	-2.34	-9.37		0.75	3	-7.02	-2.34	-9.36		0.75	3	-4.77	-1.59	-6.35
1	4	-9.28	-2.32	-9.28		1	4	-9.32	-2.33	-9.32		1	4	-5.86	-1.47	-5.86
**Ir(100)**						**Ir(110)**						**Ir(111)**				
ML	*n*H	ΔE (eV)	ΔE (eV) /H-atom	ΔE (eV) /ML		ML	*n*H	ΔE (eV)	ΔE (eV) /H-atom	ΔE (eV) /ML		ML	*n*H	ΔE (eV)	ΔE (eV) /H-atom	ΔE (eV) /ML
0.25	1	-2.83	-2.83	-11.30		0.25	1	-2.72	-2.72	-10.89		0.25	1	-2.68	-2.68	-10.70
0.5	2	-5.71	-2.85	-11.42		0.5	2	-5.45	-2.73	-10.90		0.5	2	-5.31	-2.66	-10.62
0.75	3	-8.39	-2.80	-11.19		0.75	3	-8.06	-2.69	-10.74		0.75	3	-7.92	-2.64	-10.56

1	4	-11.11	-2.78	-11.11		1	4	-10.74	-2.69	-10.74		1	4	-10.52	-2.63	-10.52
**Ni(100)**						**Ni(110)**						**Ni(111)**				
ML	*n*H	ΔE (eV)	ΔE (eV) /H-atom	ΔE (eV) /ML		ML	*n*H	ΔE (eV)	ΔE (eV) /H-atom	ΔE (eV) /ML		ML	*n*H	ΔE (eV)	ΔE (eV) /H-atom	ΔE (eV) /ML
0.25	1	-2.22	-2.22	-8.89		0.25	1	-2.21	-2.21	-8.82		0.25	1	-2.22	-2.22	-8.88
0.5	2	-4.42	-2.21	-8.84		0.5	2	-5.33	-2.66	-10.65		0.5	2	-4.28	-2.14	-8.55
0.75	3	-8.75	-2.92	-11.67		0.75	3	-8.04	-2.68	-10.72		0.75	3	-6.23	-2.08	-8.31
1	4	-11.29	-2.82	-11.29		1	4	-10.75	-2.69	-10.75		1	4	-8.10	-2.03	-8.10
**Pd(100)**						**Pd(110)**						**Pd(111)**				
ML	*n*H	ΔE (eV)	ΔE (eV) /H-atom	ΔE (eV) /ML		ML	*n*H	ΔE (eV)	ΔE (eV) /H-atom	ΔE (eV) /ML		ML	*n*H	ΔE (eV)	ΔE (eV) /H-atom	ΔE (eV) /ML
0.25	1	-2.28	-2.28	-9.12		0.25	1	-2.21	-2.21	-8.84		0.25	1	-2.30	-2.30	-9.19
0.5	2	-4.55	-2.27	-9.09		0.5	2	-4.40	-2.20	-8.80		0.5	2	-4.53	-2.26	-9.05
0.75	3	-6.72	-2.24	-8.96		0.75	3	-6.53	-2.18	-8.70		0.75	3	-6.71	-2.24	-8.95
1	4	-11.04	-2.76	-11.04		1	4	-8.64	-2.16	-8.64		1	4	-8.84	-2.21	-8.84
**Pt(100)**						**Pt(110)**						**Pt(111)**				
ML	*n*H	ΔE (eV)	ΔE (eV) /H-atom	ΔE (eV) /ML		ML	*n*H	ΔE (eV)	ΔE (eV) /H-atom	ΔE (eV) /ML		ML	*n*H	ΔE (eV)	ΔE (eV) /H-atom	ΔE (eV) /ML
0.25	1	-2.75	-2.75	-10.99		0.25	1	-2.85	-2.85	-11.42		0.25	1	-2.69	-2.69	-10.74
0.5	2	-5.52	-2.76	-11.04		0.5	2	-5.68	-2.84	-11.36		0.5	2	-5.31	-2.65	-10.61
0.75	3	-8.09	-2.70	-10.79		0.75	3	-8.46	-2.82	-11.28		0.75	3	-7.87	-2.62	-10.49
1	4	-10.69	-2.67	-10.69		1	4	-11.21	-2.80	-11.21		1	4	-10.40	-2.60	-10.40
**Rh(100)**						**Rh(110)**						**Rh(111)**				
ML	*n*H	ΔE (eV)	ΔE (eV) /H-atom	ΔE (eV) /ML		ML	*n*H	ΔE (eV)	ΔE (eV) /H-atom	ΔE (eV) /ML		ML	*n*H	ΔE (eV)	ΔE (eV) /H-atom	ΔE (eV) /ML
0.25	1	-2.47	-2.47	-9.89		0.25	1	-2.26	-2.26	-9.05		0.25	1	-2.45	-2.45	-9.79
0.5	2	-4.93	-2.46	-9.85		0.5	2	-4.51	-2.25	-9.02		0.5	2	-4.84	-2.42	-9.67
0.75	3	-7.26	-2.42	-9.68		0.75	3	-8.01	-2.67	-10.68		0.75	3	-7.18	-2.39	-9.57
1	4	-10.98	-2.75	-10.98		1	4	-10.69	-2.67	-10.69		1	4	-9.49	-2.37	-9.49

References

[1] G. Herzberg and A. Monfils, "The dissociation energies of the H_2, HD, and D_2 molecules," *Journal of Molecular Spectroscopy,* vol. 5, no. 1, pp. 482-498, 1961/01/01/ 1961, doi: https://doi.org/10.1016/0022-2852(61)90111-4.

[2] S. Vuckovic, L. O. Wagner, A. Mirtschink, and P. Gori-Giorgi, "Hydrogen Molecule Dissociation Curve with Functionals Based on the Strictly Correlated Regime," *Journal of Chemical Theory and Computation,* vol. 11, no. 7, pp. 3153-3162, 2015/07/14 2015, doi: 10.1021/acs.jctc.5b00387.

[3] F. Zhai *et al.*, "A thermodynamics study of hydrogen interaction with (1 1 0) transition metal surfaces," *Applied Surface Science,* vol. 545, 2021, doi: 10.1016/j.apsusc.2021.148961.

[4] E. d. V. Gómez, S. Amaya-Roncancio, L. B. Avalle, D. H. Linares, and M. C. Gimenez, "DFT study of adsorption and diffusion of atomic hydrogen on metal surfaces," *Applied Surface Science*, vol. 420, pp. 1-8, 2017, doi: 10.1016/j.apsusc.2017.05.032.

[5] B. W. J. Chen, D. Kirvassilis, Y. Bai, and M. Mavrikakis, "Atomic and Molecular Adsorption on Ag(111)," *The Journal of Physical Chemistry C,* vol. 123, no. 13, pp. 7551-7566, 2019/04/04 2018, doi: 10.1021/acs.jpcc.7b11629.

[6] O. M. Løvvik and R. A. Olsen, "Density functional calculations of hydrogen adsorption on palladium–silver alloy surfaces," *The Journal of Chemical Physics,* vol. 118, no. 7, pp. 3268-3276, 2003/02/15 2003, doi: 10.1063/1.1536955.

[7] P. Ferrin, S. Kandoi, A. U. Nilekar, and M. Mavrikakis, "Hydrogen adsorption, absorption and diffusion on and in transition metal surfaces: A DFT study," *Surface Science,* vol. 606, no. 7-8, pp. 679-689, 2012, doi: 10.1016/j.susc.2011.12.017.

[8] C. M. Lousada and P. A. Korzhavyi, "Hydrogen at symmetric tilt grain boundaries in aluminum: segregation energies and structural features," *Scientific Reports,* vol. 12, no. 1, p. 19872, 2022/11/18 2022, doi: 10.1038/s41598-022-23535-9.

[9] J. Paul, "Hydrogen adsorption on Al(100)," *Phys Rev B Condens Matter,* vol. 37, no. 11, pp. 6164-6174, Apr 15 1988, doi: 10.1103/physrevb.37.6164.

[10] Y. Liu, Y. Huang, Z. Xiao, and G. Jia, "First Principles Study of Adsorption of Hydrogen on Typical Alloying Elements and Inclusions in Molten 2219 Al Alloy," *Materials (Basel),* vol. 10, no. 7, Jul 19 2017, doi: 10.3390/ma10070816.

[11] Y. Liu, Y. Huang, Z. Xiao, and X. Reng, "Study of Adsorption of Hydrogen on Al, Cu, Mg, Ti Surfaces in Al Alloy Melt via First Principles Calculation," *Metals,* vol. 7, no. 1, 2017, doi: 10.3390/met7010021.

[12] J. W. Wang and H. R. Gong, "Adsorption and diffusion of hydrogen on Ti, Al, and TiAl surfaces," *International Journal of Hydrogen Energy,* vol. 39, no. 11, pp. 6068-6075, 2014, doi: 10.1016/j.ijhydene.2014.01.126.

[13] J. Greeley and M. Mavrikakis, "Surface and subsurface hydrogen: adsorption properties on transition metals and near-surface alloys," *J Phys Chem B,* vol. 109, no. 8, pp. 3460-71, Mar 3 2005, doi: 10.1021/jp046540q.

[14] L. Kristinsdóttir and E. Skúlason, "A systematic DFT study of hydrogen diffusion on transition metal surfaces," *Surface Science,* vol. 606, no. 17-18, pp. 1400-1404, 2012, doi: 10.1016/j.susc.2012.04.028.

[15] J. Strömquist, L. Bengtsson, M. Persson, and B. Hammer, "The dynamics of H absorption in and adsorption on Cu(111)," *Surface Science,* vol. 397, no. 1-3, pp. 382-394, 1998/02/01/ 1998, doi: 10.1016/s0039-6028(97)00759-0.

[16] C. M. Lousada, A. J. Johansson, and P. A. Korzhavyi, "Thermodynamics of H_2O Splitting and H2 Formation at the Cu(110)–Water Interface," *The Journal of Physical Chemistry C,* vol. 119, no. 25, pp. 14102-14113, 2015/06/25 2015, doi: 10.1021/acs.jpcc.5b01154.

[17] C. M. Lousada, A. J. Johansson, and P. A. Korzhavyi, "Molecular and dissociative adsorption of water and hydrogen sulfide at perfect and defective Cu(110) surfaces," *Phys Chem Chem Phys,* vol. 19, no. 11, pp. 8111-8120, Mar 15 2017, doi: 10.1039/c6cp07732c.

[18] C. M. Lousada, A. J. Johansson, and P. A. Korzhavyi, "Adsorption of Hydrogen Sulfide, Hydrosulfide and Sulfide at Cu(110) - Polarizability and Cooperativity Effects. First Stages of Formation of a Sulfide Layer," *ChemPhysChem,* vol. 19, no. 17, pp. 2159-2168, 2018, doi: https://doi.org/10.1002/cphc.201800246.

[19] C. M. Lousada and P. A. Korzhavyi, "Hydrogen sorption capacity of crystal lattice defects and low Miller index surfaces of copper," *Journal of Materials Science,* vol. 55, no. 15, pp. 6623-6636, 2020, doi: 10.1007/s10853-020-04459-z.

[20] P. Nordlander, S. Holloway, and J. K. Nørskov, "Hydrogen adsorption on metal surfaces," *Surface Science,* vol. 136, no. 1, pp. 59-81, 1984/01/01/ 1984, doi: 10.1016/0039-6028(84)90655-1.

[21] A. Mohsenzadeh, K. Bolton, and T. Richards, "DFT study of the adsorption and dissociation of water on Ni(111), Ni(110) and Ni(100) surfaces," *Surface Science,* vol. 627, pp. 1-10, 2014, doi: 10.1016/j.susc.2014.04.006.

[22] M. Pozzo, D. Alfe, A. Amieiro, S. French, and A. Pratt, "Hydrogen dissociation and diffusion on Ni- and Ti-doped Mg(0001) surfaces," *J Chem Phys,* vol. 128, no. 9, p. 094703, Mar 7 2008, doi: 10.1063/1.2835541.

[23] G. Kresse and J. Hafner, "First-principles study of the adsorption of atomic H on Ni (111), (100) and (110)," *Surface Science,* vol. 459, no. 3, pp. 287-302, 2000/07/10/ 2000, doi: 10.1016/s0039-6028(00)00457-x.

[24] S. Gudmundsdottir, E. Skulason, K. J. Weststrate, L. Juurlink, and H. Jonsson, "Hydrogen adsorption and desorption at the Pt(110)-(1x2) surface: experimental and theoretical study," *Phys Chem Chem Phys,* vol. 15, no. 17, pp. 6323-32, May 7 2013, doi: 10.1039/c3cp44503h.

[25] S. Hong and T. S. Rahman, "Adsorption and diffusion of hydrogen on Pd(211) and Pd(111): Results from first-principles electronic structure calculations," *Physical Review B,* vol. 75, no. 15, 2007, doi: 10.1103/PhysRevB.75.155405.

[26] F. Faglioni and W. A. Goddard, 3rd, "Energetics of hydrogen coverage on group VIII transition metal surfaces and a kinetic model for adsorption/desorption," *J Chem Phys,* vol. 122, no. 1, p. 14704, Jan 1 2005, doi: 10.1063/1.1814938.

[27] T. T. T. Hanh, Y. Takimoto, and O. Sugino, "First-principles thermodynamic description of hydrogen electroadsorption on the Pt(111) surface," *Surface Science,* vol. 625, pp. 104-111, 2014, doi: 10.1016/j.susc.2014.03.006.

[28] L. Farzaneh and A. Nakhaei Pour, "A DFT Study of Hydrogen Adsorption on Metallic Platinum: Associative or Dissociative Adsorption," *Physical Chemistry Research,* vol. 11, no. 3, pp. 527-536, 2023, doi: 10.22036/pcr.2022.339913.2089.

[29] C. D. Vurdu, "The Adsorption and Diffusion Manners of Hydrogen Atoms on Pt (100), Pt (110), and Pt (111) Surfaces," *Advances in Condensed Matter Physics,* vol. 2018, pp. 1-10, 2018, doi: 10.1155/2018/4186968.

[30] K. Christmann, "Interaction of Hydrogen With Solid Surfaces," *Surface Science Reports 9,* vol. 9, pp. 1-163, 1988.

[31] T. Usman and M.-q. Tan, "H2S adsorption and dissociation on Rh(110) surface: a first-principles study," *Adsorption,* vol. 24, no. 6, pp. 563-574, 2018, doi: 10.1007/s10450-018-9963-0.

[32] S. Wilke, V. Natoli, and M. H. Cohen, "Theoretical investigation of water formation on Rh and Pt Surfaces," *The Journal of Chemical Physics,* vol. 112, no. 22, pp. 9986-9995, 2000, doi: 10.1063/1.481645.

[33] M. Mavrikakis, J. Rempel, J. Greeley, L. B. Hansen, and J. K. Nørskov, "Atomic and molecular adsorption on Rh(111)," *The Journal of Chemical Physics,* vol. 117, no. 14, pp. 6737-6744, 2002, doi: 10.1063/1.1507104.

[34] J. E. Fisher, "Structure and magnetic anisotropy of f.c.c. cobalt films between 150° and 870 °K," *Thin Solid Films,* vol. 5, no. 1, pp. 53-60, 1970/01/01/ 1970, doi: https://doi.org/10.1016/0040-6090(70)90051-9.

[35] M. Všianská, H. Vémolová, and M. Šob, "Segregation of sp-impurities at grain boundaries and surfaces: comparison of fcc cobalt and nickel," *Modelling and Simulation in Materials Science and Engineering,* vol. 25, no. 8, p. 085004, 2017/10/27 2017, doi: 10.1088/1361-651X/aa86bf.

[36] G. Patel *et al.*, "Structural and magnetic properties of thin cobalt films with mixed hcp and fcc phases," *Physical Review B*, vol. 108, no. 18, p. 184429, 11/27/ 2023, doi: 10.1103/PhysRevB.108.184429.

[37] J. Rajeswari, H. Ibach, C. M. Schneider, A. T. Costa, D. L. R. Santos, and D. L. Mills, "Surface spin waves of fcc cobalt films on Cu(100): High-resolution spectra and comparison to theory," *Physical Review B*, vol. 86, no. 16, p. 165436, 10/22/ 2012, doi: 10.1103/PhysRevB.86.165436.

[38] F. Barbir, "Chapter Three - Fuel Cell Electrochemistry," in *PEM Fuel Cells (Second Edition)*, F. Barbir Ed. Boston: Academic Press, 2013, pp. 33-72.

[39] J. K. Nørskov *et al.*, "Trends in the Exchange Current for Hydrogen Evolution," *Journal of The Electrochemical Society*, vol. 152, no. 3, p. J23, 2005/01/24 2005, doi: 10.1149/1.1856988.

[40] D. Eberhardt, E. Santos, and W. Schmickler, "Hydrogen evolution on silver single crystal electrodes—first results1Dedicated to Professor W. Vielstich on the occasion of his 75th birthday.1," *Journal of Electroanalytical Chemistry*, vol. 461, no. 1, pp. 76-79, 1999/01/29/ 1999, doi: https://doi.org/10.1016/S0022-0728(98)00093-X.

[41] S. Trasatti, "Work function, electronegativity, and electrochemical behaviour of metals: III. Electrolytic hydrogen evolution in acid solutions," *Journal of Electroanalytical Chemistry and Interfacial Electrochemistry*, vol. 39, no. 1, pp. 163-184, 1972/09/01/ 1972, doi: https://doi.org/10.1016/S0022-0728(72)80485-6.

[42] G. N. Derry, M. E. Kern, and E. H. Worth, "Recommended values of clean metal surface work functions," *Journal of Vacuum Science & Technology A*, vol. 33, no. 6, p. 060801, 2015/11/01 2015, doi: 10.1116/1.4934685.
